# Aerobic transformation of cadmium through metal sulfide biosynthesis in photosynthetic microorganisms

**DOI:** 10.1186/1471-2180-13-161

**Published:** 2013-07-15

**Authors:** Chad D Edwards, Joseph C Beatty, Jacqueline BR Loiselle, Katya A Vlassov, Daniel D Lefebvre

**Affiliations:** 1 Department of Biology, Queen’s University, Kingston, ON, K7L 3N6, Canada

## Abstract

**Background:**

Cadmium is a non-essential metal that is toxic because of its interference with essential metals such as iron, calcium and zinc causing numerous detrimental metabolic and cellular effects. The amount of this metal in the environment has increased dramatically since the advent of the industrial age as a result of mining activities, the use of fertilizers and sewage sludge in farming, and discharges from manufacturing activities. The metal bioremediation utility of phototrophic microbes has been demonstrated through their ability to detoxify Hg(II) into HgS under aerobic conditions. Metal sulfides are generally very insoluble and therefore, biologically unavailable.

**Results:**

When Cd(II) was exposed to cells it was bioconverted into CdS by the green alga *Chlamydomonas reinhardtii*, the red alga *Cyanidioschyzon merolae*, and the cyanobacterium, *Synechoccocus leopoliensis*. Supplementation of the two eukaryotic algae with extra sulfate, but not sulfite or cysteine, increased their cadmium tolerances as well as their abilities to produce CdS, indicating an involvement of sulfate assimilation in the detoxification process. However, the combined activities of extracted serine acetyl-transferase (SAT) and *O*-acetylserine(thiol)lyase (OASTL) used to monitor sulfate assimilation, was not significantly elevated during cell treatments that favored sulfide biosynthesis. It is possible that the prolonged incubation of the experiments occurring over two days could have compensated for the low rates of sulfate assimilation. This was also the case for *S. leopoliensis* where sulfite and cysteine as well as sulfate supplementation enhanced CdS synthesis. In general, conditions that increased cadmium sulfide production also resulted in elevated cysteine desulfhydrase activities, strongly suggesting that cysteine is the direct source of sulfur for CdS synthesis.

**Conclusions:**

Cadmium(II) tolerance and CdS formation were significantly enhanced by sulfate supplementation, thus indicating that algae and cyanobacteria can produce CdS in a manner similar to that of HgS. Significant increases in sulfate assimilation as measured by SAT-OASTL activity were not detected. However, the enhanced activity of cysteine desulfhydrase indicates that it is instrumental in the provision of H_2_S for aerobic CdS biosynthesis.

## Background

Cadmium toxicity is a prevalent environmental contaminant, causing adverse effects to a wide variety of ecosystems. As a result, human-cadmium interaction has become more common, posing undesirable health effects in humans. Cadmium is a known carcinogen, and has been linked to renal failure, cellular senescence, and inhibition of essential enzymes responsible for proper cellular function [1-3]. Cadmium acts by displacing Ca(II) and Zn(II) as cofactors in numerous enzymes, and it also disrupts membrane potentials [4]. In plants and algae high concentrations of cadmium can negatively affect nitrate, phosphate and sulfate assimilation [5-8], photosynthesis [9], carbohydrate metabolism [10] and plant-water interactions [11]. Similar effects have also been shown to occur in the cyanobacterium, *Synechocystis*, where it appears that the breakdown of photosynthetic apparatus supplies nutrients for the synthesis of proteins involved in Cd tolerance [12].

Previous research has determined that photosynthetic microorganisms [13-15] and fungi [16] have the capacity to biotransform Hg(II) into metacinnabar (βHgS) under aerobic conditions. Metal sulfides possess low solubilities and, therefore, low toxicities because they are biologically unavailable. Metal biotransformation of this nature by these organisms was able to remove mercury to levels that conform to the water quality standards of the US Environmental Protection Agency. The exposure of 200 ppb Hg(II) to the red alga, *Galdieria sulphuraria*, led to the transformation of 90% of the Hg(II) into *meta*-cinnabar within 20 minutes [14].

The present study was undertaken to determine if Cd(II) is biotransformed into cadmium sulfide in a similar manner to Hg(II) under oxic conditions. This was performed in three candidate autotrophic microorganisms, the green alga *Chlamydomonas reinhardtii*, the red alga *Cyanidioschyzon merola*, and the cyanobacterium *Synechococcus leopoliensis*. Because the availability of cysteine and intermediate compounds of sulfate metabolism have been demonstrated to increase the resistance and accumulation of Cd(II) in plants [11] and protists [17], the effect of supplementation with sulfur containing compounds on cadmium sulfide synthesis was also investigated. The role of the sulfate assimilation pathway was determined by measuring the combined activities of serine acetyl-transferase (SAT, EC 2.3.1.30) and *O*-acetylserine(thiol)lyase (OASTL, EC 4.2.99.8) during Cd(II) exposure. Likewise, cysteine desulfhydrase (EC 4.4.1.1) was measured to see if cysteine could be acting as an important source of sulfide for aerobic metal biotransformation in cyanobacteria and freshwater algae.

## Results

### Cadmium tolerance in response to sulfur supplementation

**Figure 1:**
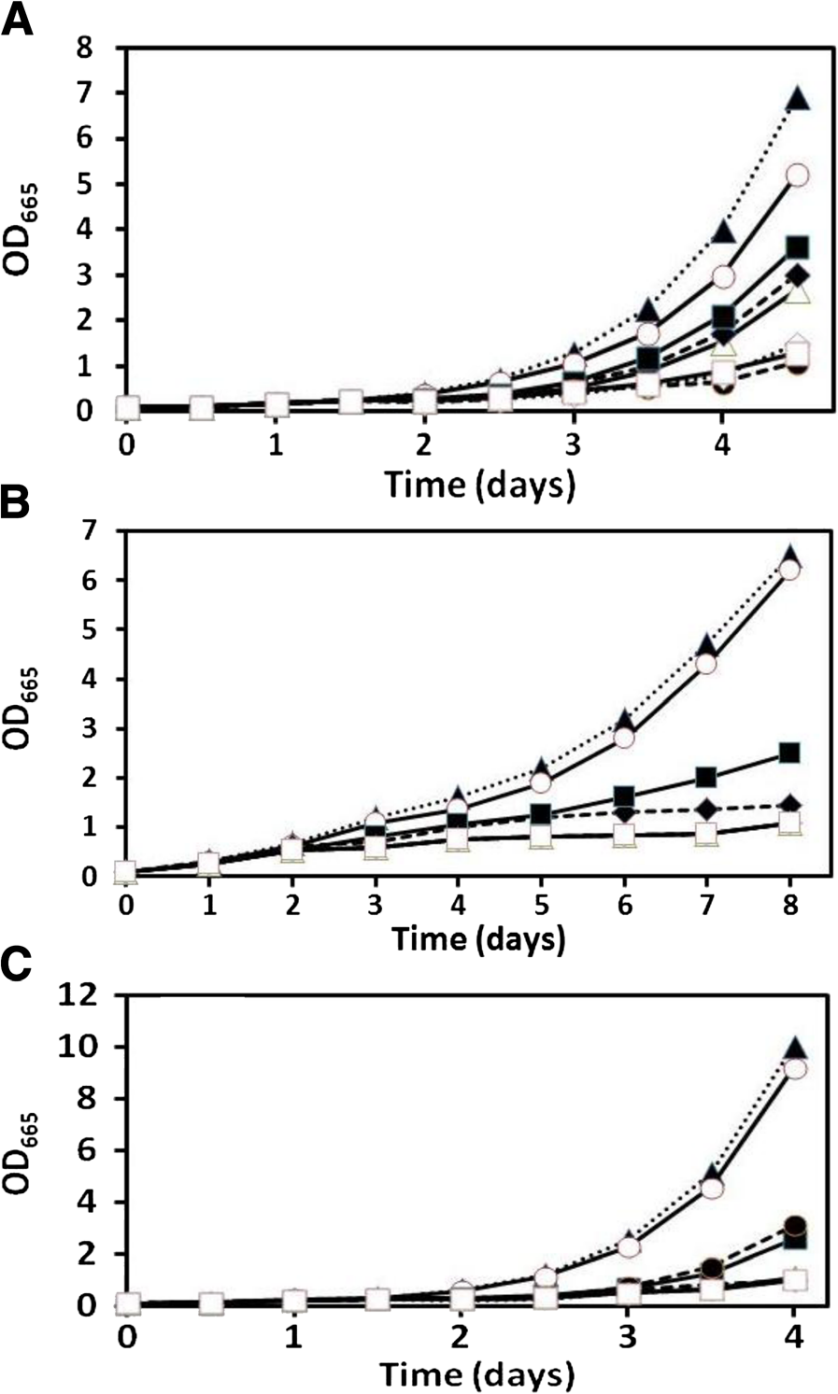
**Cadmium tolerances of *Chlamydomonas reinhardtii *(A), *Cyanidioschyzon merolae *(B), and *Synechococcus leopoliensis *(C) exposed to 100, 100, and 2 μM Cd(II), respectively, when supplemented with sulfur containing compounds.** No added Cd(II) (), Cd(II) alone (), and Cd(II) with the following additions; sulfate (), prefed sulfate plus sulfate (), sulfite (), prefed sulfite plus sulfite (), cysteine (), and prefed cysteine plus cysteine (). Means are presented (n = 4). SE always less than 7%. Where growth curves are not visible, they are at the same values as the lowest presented.

The autotrophic microalgae, *Chlamydomonas reinhardtii* and *Cyanidioschyzon merolae*, and the cyanobacterium, *Synechococcus leopoliensis*, possess a wide range of tolerances to cadmium. A concentration of Cd(II) was chosen for each species that retarded, yet did not completely inhibit, growth (Figure 1). For each of the candidate species, the provision of ten times normal sulfate prior to and during exposure to Cd ions resulted in a significant increase in growth in the cells (ANOVA, p < 0.05). In the cases of *Cyanidioschyzon* and *Synechococcus*, under this treatment, cells grew similarly to those grown in the absence of added cadmium (ANOVA, p > 0.05) whereas the *Chlamydomonas* cells grew to approx. 70% the biomass of the control. Slight increases in growth occurred during the simultaneous addition of sulfate in all species as well as in *Synechococcus* that was pre-fed and simultaneously treated with cysteine. Otherwise, treatments with sulfite and cysteine did not result in significant increases in biomass production (p > 0.05) and actually had further deleterious effects on growth as shown by similar or less growth than treatments with Cd(II) alone.

### Metal sulfide production

**Figure 2:**
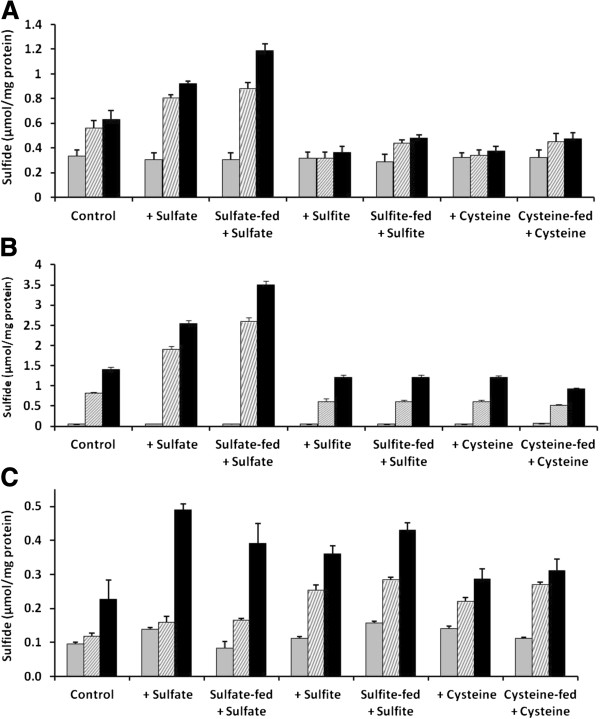
**Cadmium induced sulfide formation at 0 (grey), 24 (cross-hatched) and 48 h (black) for *Chlamydomonas reinhardtii *(A) and *Cyanidioschyzon merolae *(B) in 100 μM Cd(II), and *Synechococcus leopoliensis *(C) in 2 μM Cd(II).** Means and SE (n = 4). An asterisk indicates significantly greater than the respective Cd(II) containing control (p < 0.05).

Acid labile sulfide production was measured after 0, 1 and 2 days of metal exposure to assess the ability of *Chlamydomonas* and *Cyanidioschyzon* to bioconvert 100 μM of Cd(II) (Figure 2A, B). Similar measurements were applied to *Synechococcus* treated with 2 μM Cd(II) (Figure 2C). In all treatment conditions the highest amount of sulfide was produced by *Cyanidioschyzon*, especially when cells were supplemented with sulfate during metal exposure and even more when also pretreated with extra sulfate (Figure 2B; p < 0.05). Similar trends also occurred but not to the same degree in *Chlamydomonas* (Figure 2A; p < 0.05). The highest amounts of metal sulfide production were 3.5 (approx. 64 fold increase) and 1.2 μmol per mg protein (approx. 4 fold increase) for *Cyanidioschyzon* and *Chlamydomonas*, respectively. The cyanobacterium *Synechococcus* in the sulfate pretreated cells produced a much lower amount of metal sulfide at 0.48 μmol per mg protein (approx. 3.5 fold increase) and this required 48 h to become significantly different from the control. However, this species was exposed to only 2 μM Cd(II), one fiftieth that of the other species because it is not as tolerant to cadmium. In contrast to the two eukaryotic algal species, the cyanobacterium also made similar amounts of metal sulfides during sulfite treatments. No species made significantly more sulfide as a product of cysteine supplementation after 48 h, although *Synechococcus* did make significantly more after 24 h.

### Serine acetyltransferase and *O*-acetylserine(thiol)lyase coupled activity

**Figure 3:**
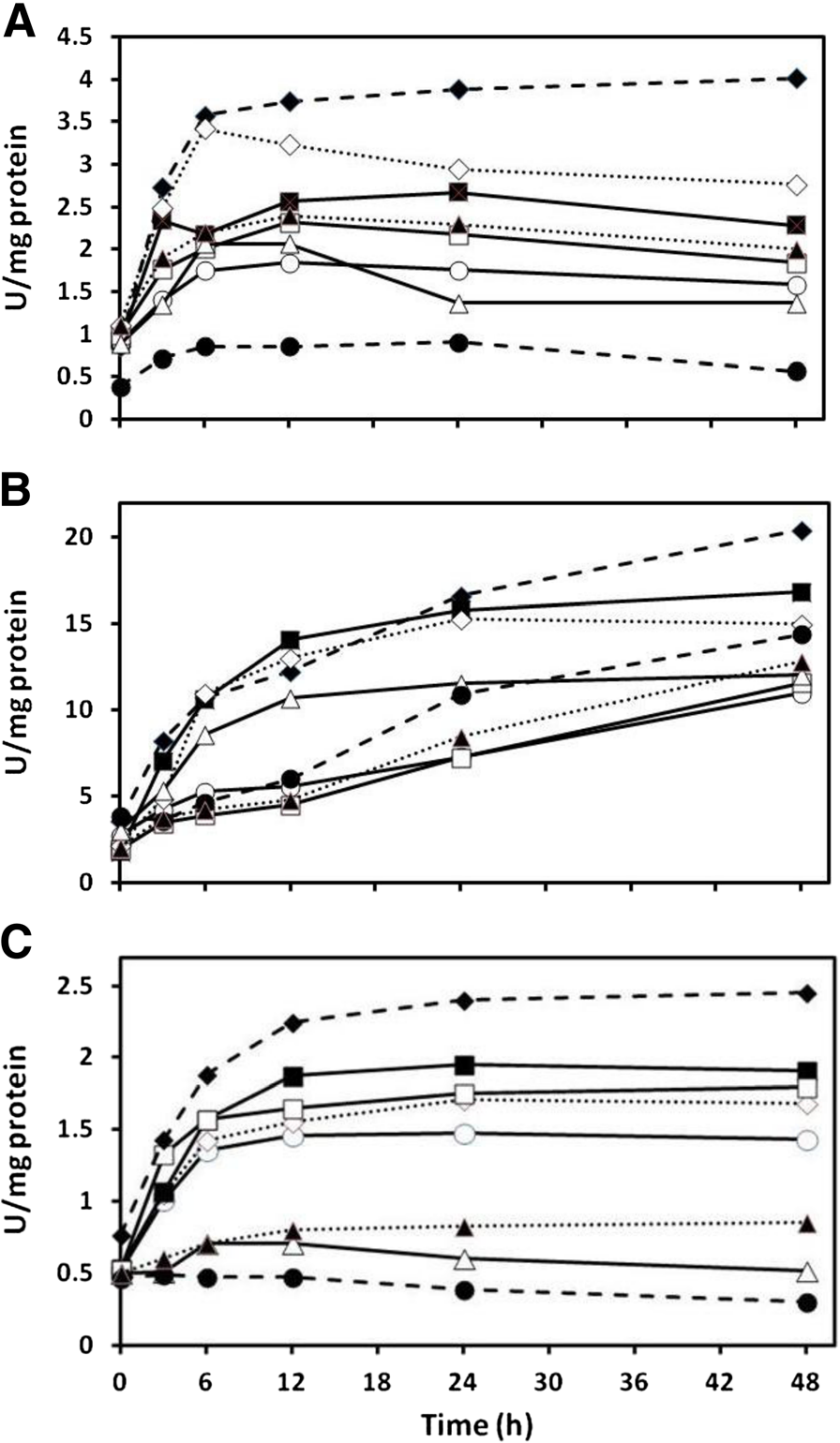
**Effect of cadmium on coupled serine acetyl-transferase and *O*-acetylserine(thiol)lyase activity in *Chlamydomonas reinhardtii *(A), *Cyanidioschyzon merolae *(B), and *Synechococcus leopoliensis *(C) exposed to 100, 100, and 2 μM Cd(II), respectively, when supplemented with sulfur containing compounds.** No added Cd(II) (), Cd(II) alone (), and Cd(II) with the following additions; sulfate (), prefed sulfate plus sulfate (), sulfite (), prefed sulfite plus sulfite (), cysteine (), and prefed cysteine plus cysteine (). Means are presented (n = 4). SE always less than 6%.

Each species had significantly different initial SAT/OASTL activities under control conditions (ANOVA, p < 0.05; Figure 3). Exposure to Cd(II) enhanced the activity of coupled SAT and OASTL over controls with no added metal after 48 hrs to 2.0, 1.7, and 3.2 fold in *Chlamydomonas* (Figure 3A), *Cyanidioschyzon* (Figure 3B), and *Synechococcus* (Figure 3C), respectively. This treatment also resulted in the highest enzyme activities in each of the species. The only other Cd(II) treatments that were higher than the controls in all three species were the simultaneously sulfate fed, and the pre- and simultaneously sulfite fed cells. The pre- and simultaneously cysteine-fed *Chlamydomonas* and *Synechococcus* had the lowest activities (ANOVA, p < 0.05), although this was not the case for *Cyanidioschyzon*. In the latter species the treatments with the lowest activities did not differ from the control, and the pre- and simultaneously cysteine-fed cells were significantly different from the control (ANOVA, p < 0.05).

Major differences between the species include the overall high SAT/OASTL activities and the relatively high pre- and simultaneously cysteine-fed treatment in *Cyanidioschyzon* and the relatively low pre- and simultaneous cysteine-fed treatment in *Chlamydomonas* and *Synechococcus*.

### Cysteine desulfhydrase

**Figure 4:**
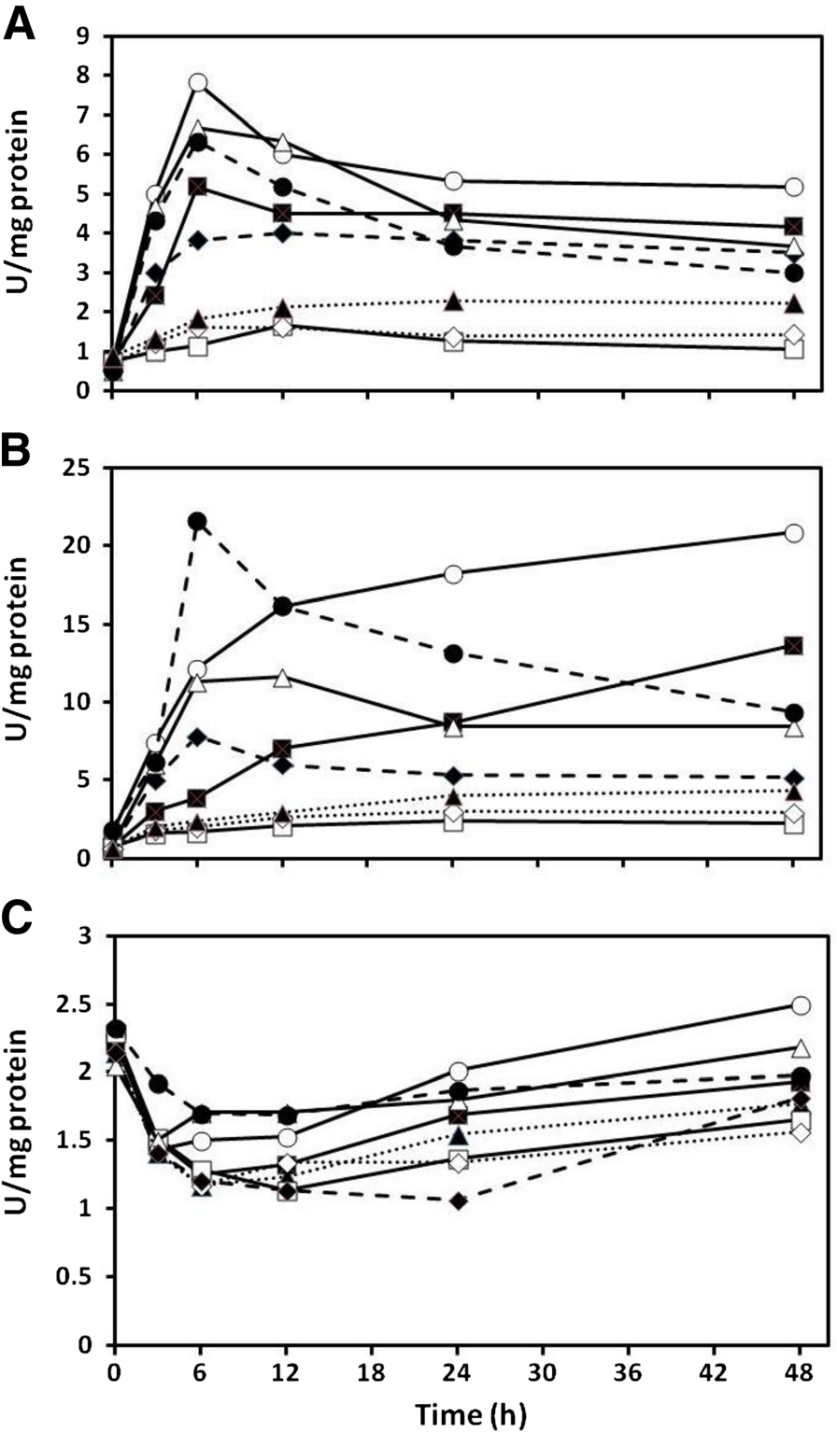
**Effect of cadmium on cysteine desulfhydrase activity in *Chlamydomonas reinhardtii *(A), *Cyanidioschyzon merolae *(B), and *Synechococcus leopoliensis *(C) exposed to 100, 100, and 2 μM Cd(II), respectively, when supplemented with sulfur containing compounds.** No added Cd(II) (), Cd(II) alone (), and Cd(II) with the following additions; sulfate (), prefed sulfate plus sulfate (), sulfite (), prefed sulfite plus sulfite (), cysteine (), and prefed cysteine plus cysteine (). Means are presented (n = 4). SE always less than 7%.

The presence of Cd(II) increased cysteine desulfhydrase activity over that of the metal free control in only one of the three investigated species, *Chlamydomonas* (Figure 4). However, of the Cd(II) treatments the pre- and simultaneously sulfate fed cells had the highest activity in all species after 48 h (ANOVA, p < 0.05). Under these conditions, *Cyanidioschyzon* had the highest cysteine desulfhydrase activity after 48 h at 21.5 U/mg protein, followed by *Chlamydomonas* at 7.8 U/mg protein, and *Synechococcus* at only 2.5 U/mg protein. Simultaneous metal and sulfate treatments consistently had the second highest final activities in the eukaryotic species, whereas for *Synechococcus*, it was the simultaneous cysteine treatment. All of the *Chlamydomonas* and *Cyanidioschyzon* treatments started with an increase in activity whereas cysteine desulfhydrase activity actually initially decreased in all *Synechococcus* cultures (Figure 4C) followed by slow recoveries up to 48 h (Figure 4C). In the eukaryotic species, both types of cysteine treatments gave transient increases with peak activities at 6 h followed by decreases in activity. All sulfide treatments resulted in relatively low cysteine desulfhydrase activities.

## Discussion

Our previous studies on Hg(II) biotransformation have shown that it can be converted into metacinnabar (HgS) in both eukaryotic algae [14] and prokaryotic cyanobacteria [15]. However, these studies did not investigate supplementation with sulfur containing compounds, nor did they assess metal sulfide production in response to Cd(II) exposure. Cadmium is also a Group 12 stable metal that is very toxic and widely distributed in the environment.

### Tolerance to Cd(II)

The levels of Cd(II) tolerance of the algae and cyanobacteria were similar to previous studies [18], and the addition of sulfate to all of the investigated species both prior to and during metal exposure resulted in higher tolerance to Cd(II) than any other treatment (Figure 1). These cultures were either the same as (*Cyanidioschyzon* and *Synechococcus*) or only slightly lower in biomass (*Chlamydomonas*) over the 48 h growth period by comparison to the metal-free controls. Although cadmium stress has been shown to induce sulfur limiting conditions [7,19], this was not entirely alleviated by the simultaneous provision of sulfate in any of the studied species, thus indicating that established metabolic reserves of sulfur other than sulfate itself, may be involved in cellular protection. Furthermore, it has been demonstrated that Cd exposure triggers a decline of photosynthetic apparatus thereby liberating sulfur as well as nitrogen and iron, which can be subsequently used for the synthesis of Cd detoxification enzymes [12]. Assimilated sulfate appears to create an organic sulfur pool that can be readily employed to biotransform Cd(II) as it enters the cell in a similar manner to that proposed for Hg(II) where chemical modification of thiols severely lessened HgS production [14,15]. Why this cannot be provided by simultaneous sulfate provision is likely to be a product of the high energy demand (732 kJ mol^-1^) required to reduce sulfate to sulfide for thiol production, energy required for sulfate uptake, and the decline in sulfate uptake induced by cadmium itself [12]. These organisms rely on photosynthesis to generate reducing power that is essential for carbon fixation. If this is shunted towards sulfate assimilation, it would inhibit cellular metabolism and growth. By temporally displacing energy requirements to a pretreatment period, this is overcome and the cells are able to adequately cope with any stress imposed by subsequent exposure to Cd(II). The simultaneous sulfate and metal treated cells grew marginally better than the cells treated with metal alone in *Cyanidioschyzon* and *Synechococcus* (Figures 1B & C), but not in *Chlamydomonas*. Metabolic differences might ac-count for this; *i.e.* the former species may have relatively more efficient sulfate assimilation. Interestingly, in a separate study it was revealed that *Synechococcus* is able to utilize elemental sulfur as a sulfur source resulting in enhanced metal tolerance (data not shown). These results point to the importance of sulfur nutrition in cadmium tolerance that has implications for other organisms [20,21], including humans [22]. Nevertheless, this has not been well documented in the literature.

The other treatment in which *Synechococcus* grew better than in cadmium alone was that in which cysteine was supplied both prior to and during metal exposure. However, this cannot be accounted for by a relatively high cysteine desulfhydrase activity in *Synechococcus* (Figure 4). Both eukaryotic species were not as adept at coping with this form of sulfur supplementation.

In general, pretreatments with sulfite, an intermediate of the sulfate assimilation pathway, and with the amino acid, cysteine, did not provide ameliorative effects on growth during Cd(II) exposure to the same degree as sulfate. Sulfite can be toxic to green algae [23] because of interactions with sulfide bonds of glutathione and glutathione disulfide that severely affect anti-oxidation processes [24]. It can also lead to SO_2_ toxicity through sulfoxy-free radicals generated by the oxidation of SO_3_^2-^ by O_2_^−^[23]. Furthermore, in membrane preparations of cyanobacteria, sulfite stimulates ATP hydrolysis and inhibits ATP synthesis [25]. Exogenous cysteine is believed to have direct effects on transporters and enzymes that are sensitive to thiol/disulfide redox variations [26]. This could account for the deleterious effects on the eukaryotic organisms in this study as unfortunately, these treatments did not improve Cd(II) tolerance. However, cysteine did improve the growth of *Synechococcus* in the presence of cadmium. It is possible that this organism is not as susceptible to functional interference of its protein thiol groups, or that it has a greater absorption and storage capacity for cysteine, thereby lowering its deleterious effects.

### Cellular sulfide production

The measurement of acid labile sulfide is a convenient way to estimate amounts of metal sulfide within samples [27]. Our studies clearly indicated that the addition of Cd(II) caused *de novo* aerobic synthesis of metal sulfide, assumed to be predominantly CdS because there was no detected increase in metal sulfides when Cd(II) was not supplied to the cells under any conditions (data not shown). This production of metal sulfide was generally comparable to that of HgS in our previous studies [13-15], and it was produced to a higher level in the more rapidly growing eukaryotic cell treatments (Figure 2A & B). The cyanobacterium, *Synechococcus*, was able to synthesize significantly higher amounts of metal sulfide over time under all investigated conditions, although it is much less tolerant to Cd(II) than the eukaryotic species. Heavy metals are known to bind with low molecular weight thiol compounds such as glutathione and phytochelatins [28,29]. The latter are low molecular weight metallothioneins synthesized from glutathione [17]. Like metal sulfides, *per se*, metals bound in this way are more stable and less likely to cause oxidative damage. Cytosolic fractions taken from species of cyanobacteria and algae after exposure to Cd(II) have shown that approximately 30% of these metals are bound with metallothioneins, including phytochelatins [30-32]. Metallothioneins can exist as low and high molecular weight variants. In low molecular weight forms the metal is bound to thiol groups, whereas in the high molecular weight forms, additional inorganic sulfur is incorporated into the complexes [33] which appear to stabilize and improve detoxification. Interestingly, it is this pool of inorganic sulfur that is probably associated with Cd to form CdS.

Sulfate treatments resulted in more production of cellular metal sulfide by comparison with the treatment with Cd(II) alone in all investigated species (Figure 2). This took longer to become apparent in the cyanobacterial species (48 h, Figure 2C) where significant differences from the control also occurred in the sulfite and cysteine treatments. The latter was not the case for *Chlamydomonas* or *Cyanidioschyzon*. Here again, this could be accounted for by sulfur metabolism differences between cyanobacteria and algae, or possibly distinct tolerances to the toxic effects of these metabolites. High rates of sulfite assimilation into amino acids [34] and high expression of *SSU1*, a sulfite efflux gene [35], are known to result in lower toxicity to sulfite in yeast. Similar mechanisms may also occur in *Synechococcus*.

The thermophilic red microalga, *Cyanidioschyzon*, was capable of biotransforming approximately three times as much Cd(II) into metal sulfide as the mesophilic green alga, *Chlamydomonas*, when both were grown in 100 μM Cd(II). This ability may be accounted for by its adaptation to sulfur-rich hot springs [36]. In fact, the *Cyanidium* medium [37] used to grow *Cyanidioschyzon* contains over an order of magnitude more sulfate than the high salt medium conventionally used for *Chlamydomonas*. The sensitivity of *Synechococcus* to Cd(II) is much higher than in the eukaryotic species. Nevertheless, metal biotransformation into sulfide by this species was only about half of that for *Chlamydomonas*, indicating that although sensitive to cadmium, it was able to transform a high proportion of the Cd(II) into metal sulfide. The fact that *Synechococcus* can convert a relatively high amount of Cd(II) into metal sulfide while remaining very sensitive to Cd(II), might be attributed to a relatively high susceptibility to displacement of metals by Cd as cofactors in photosynthetic and other metabolic enzymes, and to disruption of membrane function [4]. Similarly, this could account for the differences between the algal species.

The first report of acid labile sulfide in living organisms was in association with metallothioneins and phytochelatins in fission yeast [38], and it is known that metallothionein gene amplification can confer resistance to cadmium in *Synechococcus* PCC 6301 [39]. Algal phytochelatins bind cadmium in relatively low metal to peptide amounts [40] and it is likely that CdS formed in the organisms in the present study are mainly in the form of precipitated nanoparticles, examples of which have been reported in as diverse organisms as *Klebsiella*[41], marine microalgae [33], tomatoes [42] and mustard plants [43]. This, however, remains to be confirmed.

### Sulfate assimilation

Most organisms absorb sulfur from the environment in the form of inorganic sulfate and active transport systems for sulfate uptake have been investigated extensively in algae [44-46], bacteria [47], yeast [48], and higher plants [49,50].

Algae and cyanobacteria appear to undergo sulfur assimilation in a similar manner [51,52]. Absorbed sulfate is first converted to adenylylsulfate (APS) by ATP sulfurylase. Adenylylsulfate is then further reduced by APS reductase to yield sulfite which in turn is converted to sulfide by sulfite reductase. This sulfide is immediately transferred to the serine acetyltransferase/*O*-acetylserine(thiol)lyase bi-enzymatic complex (SAT-OASTL) that covalently binds it to serine to produce cysteine [50,51]. Because all assimilated sulfate is converted into cysteine via SAT-OASTL, measuring these enzymes’ coupled activity provides a convenient means of comparing sulfate assimilation between species in response to various treatments.

The activities of SAT-OASTL in *Chlamydomonas* were similar to those of Ravina and colleagues [52] in the non-metal controls. In addition, their sulfite treatment had a similar activity to the pre- and simultaneously fed sulfite treatment in the present study. However, it is difficult to assess the effect of sulfite on specific enzymes because of its cellular toxicity (Figure 1A), something that was not considered in the previous study. The highest enzyme activities occurred when Cd(II) was provided without any supplemental sulfur containing compounds, a state in which sulfur reserves of the cells would be consumed in the CdS synthetic process (Figure 2A). Sulfur starvation has been previously shown to significantly up-regulate OASTL activity [52] as has Cd(II) exposure ([5], but this has never been studied in the context of aerobic cadmium sulfide synthesis. The highest bioconversion of Cd(II) into metal sulfide was performed when *Chlamydomonas* was supplemented with extra sulfate. However, this did not result in significant differences in SAT-OASTL activity from the non-metal control which was significantly lower than the Cd(II) control. This could be because Cd-elicited sulfur deprivation in the cells is compensated for by sulfate provision.

Similar to *Chlamydomonas*, both *Cyanidioschyzon* and *Synechococcus* possessed the highest SAT-OASTL activities during the Cd(II) control conditions. However, unlike in *Chlamydomonas*, simultaneous sulfate treatments had significantly higher activities than the non-metal controls (ANOVA, p < 0.05). This appears to be contradictory because these cells have higher S-nutrition than the controls and it has been shown that S-deprivation enhances OASTL activity [52]. However, Cd-induced S-deprivation does not appear to be compensated for by the simultaneous provision of sulfate whereas extra sulfate provided by additional pre-treatments did lower enzyme activity to closer to the control levels, thereby revealing an S-nutritional effect.

Major differences occurred in the cysteine treatments between *Chlamydomonas* and *Synechococcus* that displayed expected low activities compared to controls, and the higher activities observed in *Cyanidioschyzon*. This could be explained by the latter species’ adaptation to sulfur-rich hot spring environments [36,53,54], as its SAT-OASTL activities were generally a magnitude higher than in the other species. *Cyanidioschyzon* enzymes need not be regulated as stringently as for *Chlamydomonas* and *Synechococcus* given that sulfur would never normally become limiting in its native environment where it could utilize the sulfur assimilation pathway for metal detoxification without experiencing the threat of sulfur depletion. Lending support to this notion is that this red alga possesses one additional SAT and two additional OASTL homologues [55]. However, it synthesized more CdS only under sulfate-, and not sulfite- or cysteine-supplemented conditions in a similar manner to *Chlamydomonas*, and in contrast to *Synechococcus* where all conditions gave significant increases in acid labile sulfide production (Figure 2C).

The extracted activity of SAT-OASTL indicates that these enzymes do play a role in the production of required assimilated sulfur for cadmium sulfide as it was highest when cells were exposed to Cd(II) without sulfate supplementation. Higher plants that have been genetically engineered to have higher levels of these enzymes have shown some increased resistance to Cd(II) [11,56] and other metals [57]. Bearing in mind that *in vivo* activity would be distinct from extracted activity because of, among other things, different substrate concentrations, it is likely that sulfur flux through SAT-OASTL would be higher in the sulfate supplemented cells, which could contribute to the respective elevated CdS production.

### Enzymatic sulfide production

Hydrogen sulfide, traditionally considered a toxic compound, has recently been implicated in cellular signaling [58,59]. However, it would be expected that metal sulfide biosynthesis should require more sulfide than signaling processes. Several metabolic sources of sulfide have been proposed [60] and of these, cysteine desulfhydrase activity is the most evident and is accentuated by feeding with cysteine [61]. In addition, there is some evidence that sulfide is released during excess sulfate nutrition which can be through provision of sulfate or sulfur dioxide/sulfite [62]. This appears to be because of inadequate cellular supplies of *O*-acetylserine [63] and as such, OASTL cannot utilize all the H_2_S generated by sulfite reductase. This could have occurred in the sulfate supplemented cultures of this study, particularly in the case of *Cyanidioschyzon* where the sulfate concentration was 108.6 mM, however sulfate in the media of the other two species was relatively low. Other metabolic sources of significant amounts of H_2_S are speculative.

The assayed activity of cysteine desulfhydrase was generally much higher in *Cyanidioschyzon* than in *Chlamydomonas* and *Synechococcus* (Figure 4) as was the case for SAT-OASTL (Figure 3). This further indicates adaptation to high sulfur environments that accounts for its elevated ability to biotransform Cd(II) into metal sulfide which is insoluble and therefore, non-toxic. The highest activity of all was at only 6 h in the pre- and simultaneous treatment of *Cyanidioschyzon* with cysteine, but this did not result in an enhanced production of metal sulfide. In fact, both types of cysteine treatments in all species had relatively high cysteine desulfhydrase activities at 6 h with no enhanced metal sulfide production. Unfortunately, treatments with lower amounts of cysteine did not result in detectable increases in metal sulfide production (data not shown). This implies that the enzyme may not be involved in the supply of sulfide for CdS synthesis, or that excess cysteine is inhibitory. The latter is likely because supplementation with sulfate prior to and during Cd(II) exposure resulted in the highest desulfhydrase activities after 24 h in all three species as well as the highest production scenarios for metal sulfide. In addition, the simultaneous addition of extra sulfate with Cd(II) also resulted in relatively high extracted enzyme activity. This is consistent with the fact that *Escherichia coli* genetically engineered to contain unregulated cysteine desulfhydrase do produce elevated amounts of CdS [64,65], and the formation of CdS nanoparticles appears to increase with extractable cysteine desulfhydrase activity in the photosynthetic bacterium *Rhodopseudomonas palustris*[66]. Although the accumulation of acid labile sulfide is high in the organisms presented in this study, it remains to be seen if they comprise CdS nanoparticles.

## Conclusions

The fact that cadmium tolerance was significantly enhanced by sulfate supplementation is supported by the discovery of the enhanced formation of metal sulfides under these conditions. Because Cd(II) was provided in the media in a much higher excess than other metal ions, the increase in acid labile sulfides can be attributed to CdS formation. The cyanobacterium *Synechococcus leopoliensis* , the green alga *Chlamydomonas reinhardtii*, and especially the red alga *Cyanidioschyzon merolae* produce high quantities of CdS in a manner that appears to be similar to HgS biosynthesis ([13-15]. The addition of sulfate increased this production dramatically indicating the involvement of sulfate assimilation. Although SAT-OASTL was not shown to increase significantly under sulfate supplementation, the relatively long-term duration of this study could account for the accumulation of reserves used to make the sulfide moiety of CdS. The identity of these reserves could be glutathione or possibly sulfur mobilized from the breakdown of photosynthetic apparatus [12]; however, this remains to be determined. Whereas the role of SAT-OASTL appears to be pedestrian, cysteine desulfhydrase can be implicated in the production of CdS because it does possess elevated activity during conditions conducive to metal sulfide production.

## Methods

### Culture sources and growth conditions

The eukaryotic alga *Chlamydomonas reinhardtii* (UTEX 90) was obtained from the Culture Collection of Algae, University of Texas at Austin. Cultures were grown in high salt medium (HSM) [67] composed of 9.35 mM NH_4_Cl, 8.27 mM K_2_HPO_4_, 5.44 mM KH_2_PO_4_, 0.09 mM CaCl_2_, 0.081 mM MgSO_4_∙7H2O, 3 μM H_3_BO_3_, 2.1 μM MnCl_2_∙4H_2_O, 1 μM Na_2_EDTA∙2H_2_O, 0.6 μM FeCl_3_∙6H_2_O, 0.03 μM NaMoO_4_∙2H_2_O , 0.025 μM ZnCl_2_, , 0.01 μM CoCl_2_∙6H_2_O, 0.07 nM CuCl_2_∙2H_2_O in double deionized water. *Cyanidioschyzon merolae* 10D was acquired from the Microbial Culture Collection of the National Institute for Environmental Studies (Tsukuba, Japan). *Cyanidioschyzon* was propagated using a *Cyanidium* medium [37] composed of 9.85 mM (NH_4_)_2_SO_4_, 2.06 mM K_2_HPO_4_, 1.01 mM MgSO_4_∙7H_2_O, 0.67 mM CaCl_2_, 13 μM Na_2_EDTA, 3.0 μM H_3_BO_3_, 2.2 μM FeCl_3_^.^6H_2_O, 1.2 μM MnCl_2_∙4H_2_O, 0.32 μM CuSO_4_∙5H_2_O, 0.22 μM ZnSO4∙7H_2_O, 0.12 μM Na_2_MoO_4_ and 0.05 μM CoCl_2_^.^6H_2_O in double deionized water. The medium was adjusted to pH 3.5 with HCl. *Synechococcus leopoliensis* (UTEX 2434), a cyanobacteria species, was obtained from the Culture Collection of Algae, University of Texas at Austin. Cells were grown in medium using 50X Cyanobacteria BG-11 Freshwater Solution (Sigma Aldrich, catalogue # C3061) [68] that was diluted to 1X in double deionized water to final concentrations of: 17.65 mM NaNO_3_, 0.3 mM MgSO_4_∙7H_2_O, 0.24 mM CaCl_2_∙2H_2_O, 0.18 mM K_2_HPO_4_, 46.0 μM H_3_BO_3_, 31 μM citric acid, 21 μM ferric ammonium citrate, 9.1 μM MnCl_2_∙4H_2_O, 2.8 μM MnNa_2_EDTA, 1.7 μM NaMoO_4_∙2H_2_O, 0.77 μM ZnSO_4_∙7H_2_O, 0.32 μM CuSO_4_∙5H_2_O, 0.17 μM Co(NO_3_)_2_∙6H_2_O. All chemicals were obtained from Sigma-Aldrich (Oakville, Canada) or Fisher Scientific (Ottawa, Canada). *Synechococcus* and *Chlamydomonas* were grown in 1.0 L of their respective media in 1.5 L Pyrex glass cylindrical bioreactors under fluorescent lighting of 150 μE /m^2^/s at 28°C. Cells were kept suspended by aerating at a 1 L per min flow rate. *Cyanidioschyzon* was grown similarly except that the temperature was maintained at 45°C [53].

### Cell treatments

The effect of sulfur nutrition on heavy metal resistance and biotransformation was investigated by exposing each species to supplemental sulfur treatments. Supplemental sulfur was provided in the form of sulfate, sulfite or cysteine. Sulfate and sulfite were added as K_2_SO_4_ and K_2_SO_3_, respectively, at ten-fold the amount of sulfur equivalents in the original media and the L-cysteine treatments were supplemented to twice the original amount of sulfur equivalents in the media. Experimental treatments included 1) no additional sulfur containing compounds, 2) additional sulfur containing compound, and 3) additional sulfur containing compound both before (pre-fed) and during the treatment period (plus). All treatments were performed in 100 mL of medium in 150 mL glass plant tissue culture vessels with translucent magenta B-caps obtained from Sigma-Aldrich (Oakville, Canada). Continuous fluorescent illumination was at 150 μE / m^2^/ s with 120 rpm rotary shaking. Culturing temperatures were 27°C for *Synechococcus* and *Chlamydomonas*, and 45°C for *Cyanidioschyzon*. The initial cell density for all cultures was O.D._665_ = 0.1. These were grown to an O.D._665_ = 1.0 and diluted tenfold with fresh media prior to metal treatments. Metal treatments were then performed in one hundred mL cell cultures in 150 mL glass cell culture jars, to which Cd(II) was added from a 25 mM CdCl_2_ stock solution. A metal ion concentration was selected for each species that slowed but did not stop growth. Cell growth was measured at O.D._665_ using a Spectra Max Plus Spectrophotometer (Molecular Devices, Sunnyvale, CA).

### Sulfide analysis

Analysis of acid labile sulfide was performed using a modified version of the protocol developed by Siegel [27]. One hundred microliter samples from the cell cultures were transferred into 1.5 mL microcentrifuge tubes. To this was added 100 μL 0.02M *N*,*N*-dimethyl-*p*-phenylenediamine sulfate in 7.2 N HCl and 100 μL of 0.3 M FeCl_3_ in 1.2 N HCl. Parafilm was used to seal the microcentrifuge caps, followed by incubation in the dark for 20 min. and centrifugation at 10,000 × g for 10 min. at room temperature. Two hundred microliters of supernatant was then transferred into the wells of a 96 well plate and optical density was measured at 670 nm using a Spectra Max Plus Spectrophotometer. Concentrations were determined by comparing results to standard curves developed with Na_2_S standards.

### Enzyme assays

Ten millilitre samples were removed from 100 mL cultures at intervals of 0, 6, 12, 24 and 48 h, transferred into 15 mL screw capped polypropylene centrifuge tubes (VWR 21008–089) and centrifuged at 3,000 *g* for 10 minutes at 4°C. The supernatant was removed, and the pellets were gently resuspended in 1 mL of ice cold 10 mM potassium phosphate buffer (pH 7.5) [69] and transferred to 1.5 mL microfuge tubes. Then, 0.05 g of 0.1 mm glass beads were added to each tube followed by homogenization for 5 minutes at maximum speed using a Bullet Blender (Next Advance, Averill Park, NY) . Homogenized samples were then frozen in liquid nitrogen and stored at −80°C until required.

The serine acetyl-transferase (SAT) and *O*-acetylserine(thiol)lyase (OASTL) combined enzyme assay was modified from Dominguez *et al.*[5]. One hundred microliters of cellular lysate was added to a 1.5 mL microcentrifuge tube, along with 20 μL of 100 mM potassium phosphate buffer (pH 7.3). Then, 9.5 μL of 400 mM L-serine was added to the reaction tube followed by 6.75 μL of 400 mM acetyl coenzyme A, 10 μL of 100 mM Na_2_S and 72 μL of double deionized water. The samples were immediately mixed by vortexing and incubated at 30°C for 20 min. The reaction was then terminated through the addition of 25 μL of 25% trichloroacetic acid. The L-cysteine produced was measured by transferring 200 μL of the sample into 5 mL test tubes containing 0.2 mL of 99.5% acetic acid ninhydrin reagent. The ninhydrin reagent was composed of 250 mg ninhydrin in 6 mL glacial acetic acid and 4 mL concentrated HCl made daily. This was mixed for 30 minutes in the dark at room temperature before use. The test tubes were then placed into a 100°C water bath for 10 min followed by rapid cooling in wet ice. The ninhydrin reaction was terminated by the addition of 1.4 mL of 99% ethanol. Two hundred microliter samples were then read on a Spectra Max Plus Spectrophotometer at 560 nm and concentrations determined by comparison with cysteine standards. Enzymatic activities are presented on a per protein basis.

Cysteine desulfhydrase activity was determined by following a modified protocol from Chu and colleagues [69]. One hundred microliter samples in 10mM potassium phosphate buffer were transferred to 1.5 mL microcentrifuge tubes. The reactions were initiated by the addition of 900 μL 0.11 mM L-cysteine followed by vortexing and incubated at 37°C for 1 h. Sulfide production was quantified by following the protocol described above in the sulfide analysis section [27].

### Protein assays

Bradford assays were determined by following the protein microplate bioassay procedure supplied by Bio-Rad (Mississauga, Canada). Protein Assay Dye Reagent concentrate was diluted 5 times in distilled water. Ice-cold samples were homogenized using a Bullet Blender (Next Advance, Averill Park, NY) for 5 minutes on its maximum speed. The homogenized cells were then transferred into fresh 1.5 mL microcentrifuge tubes and centrifuged at 1000 *g* for 5 min to pellet cellular debris. Then 80 μL samples from the supernatant were diluted with 720 μL of double deionized water. To this 200 μL of dye reagent was added to each tube, vortexed and the samples incubated at room temperature for 5 minutes. Two hundred microliter aliquots were then read at 595 nm in a Spectra Max Plus Spectrophotometer.

### Statistics

Analysis of variance (ANOVAS) and Tukey-Kramer post hoc tests were performed using JMP 8.0 software (SAS Incorporated.), or where appropriate, T-tests were analyzed using Microsoft Excel 2007. All experiments include representative standard errors (SE). Experiments were performed at least in triplicate and the results are indicative of n = 3 for enzymatic assays. SE is presented in all figures by the error bars. Where it is not visible, SE is smaller than the character at that point.

## Competing interests

The authors declare that they have no competing interests.

## Authors’ contributions

CDE: metal tolerance analysis, sulfide measurements, enzyme activity analysis, interpretation of data, manuscript suggestions. JCB: cell culture of *Chlamydomonas*, analysis of cysteine desulfhydrase activity. JBRL: cell culture of *Cyanidioschyzon*, sulfide and enzyme activity analysis. KAV: cell culture of *Synechococcus*, sulfide and enzyme activity analysis. DDL: conception and design, supervision of the research group, funding support, drafting and revising the manuscript. All authors approved the final manuscript.
